# Role of Microbiota-Derived Hydrogen Sulfide (H_2_S) in Modulating the Gut–Brain Axis: Implications for Alzheimer’s and Parkinson’s Disease Pathogenesis

**DOI:** 10.3390/biomedicines12122670

**Published:** 2024-11-23

**Authors:** Constantin Munteanu, Gelu Onose, Mariana Rotariu, Mădălina Poștaru, Marius Turnea, Anca Irina Galaction

**Affiliations:** 1 Department of Biomedical Sciences, Faculty of Medical Bioengineering, University of Medicine and Pharmacy “Grigore T. Popa”, 700454 Iasi, Romania; madalina.postaru@umfiasi.ro (M.P.); marius.turnea@umfiasi.ro (M.T.); anca.galaction@umfiasi.ro (A.I.G.); 2 Neuromuscular Rehabilitation Clinic Division, Clinical Emergency Hospital “Bagdasar-Arseni”, 041915 Bucharest, Romania; gelu.onose@umfcd.ro; 3 Faculty of Medicine, University of Medicine and Pharmacy “Carol Davila”, 020022 Bucharest, Romania

**Keywords:** microbiota, dysbiosis, H_2_S, gut–brain axis, Alzheimer’s disease, Parkinson’s disease, glial cell dysfunction, neuroinflammation, neurodegeneration, probiotics, prebiotics

## Abstract

Microbiota-derived hydrogen sulfide (H_2_S) plays a crucial role in modulating the gut–brain axis, with significant implications for neurodegenerative diseases such as Alzheimer’s and Parkinson’s. H_2_S is produced by sulfate-reducing bacteria in the gut and acts as a critical signaling molecule influencing brain health via various pathways, including regulating inflammation, oxidative stress, and immune responses. H_2_S maintains gut barrier integrity at physiological levels and prevents systemic inflammation, which could impact neuroinflammation. However, as H_2_S has a dual role or a Janus face, excessive H_2_S production, often resulting from gut dysbiosis, can compromise the intestinal barrier and exacerbate neurodegenerative processes by promoting neuroinflammation and glial cell dysfunction. This imbalance is linked to the early pathogenesis of Alzheimer’s and Parkinson’s diseases, where the overproduction of H_2_S exacerbates beta-amyloid deposition, tau hyperphosphorylation, and alpha-synuclein aggregation, driving neuroinflammatory responses and neuronal damage. Targeting gut microbiota to restore H_2_S homeostasis through dietary interventions, probiotics, prebiotics, and fecal microbiota transplantation presents a promising therapeutic approach. By rebalancing the microbiota-derived H_2_S, these strategies may mitigate neurodegeneration and offer novel treatments for Alzheimer’s and Parkinson’s diseases, underscoring the critical role of the gut–brain axis in maintaining central nervous system health.

## 1. Introduction

The human gastrointestinal (GI) tract harbors a complex community of trillions of microorganisms, including bacteria, archaea, fungi, and viruses, collectively referred to as the gut microbiota [1]. These microorganisms are crucial for maintaining homeostasis and performing essential functions such as digestion, nutrient absorption, immune system regulation, and the maintenance of the intestinal barrier [2]. In recent years, a growing body of evidence has highlighted the role of gut microbiota beyond the GI tract in modulating brain functions and contributing to the onset and progression of neurodegenerative diseases [3]. The gut and brain interaction, called the gut–brain axis, is a bidirectional communication system involving neural, immune, and endocrine pathways [4]. This system ensures proper gut and brain functioning and is significantly influenced by microbial metabolites such as short-chain fatty acids (SCFAs), tryptophan metabolites, and hydrogen sulfide (H_2_S).

**Figure 1:**
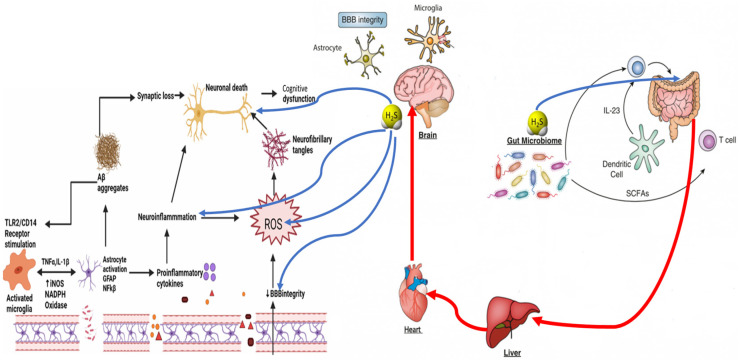
The dysbiosis-associated overproduction of H_2_S is proposed as a contributing factor in the progression of neurodegenerative diseases, where neuroinflammation plays a pivotal role. At the same time, H_2_S can have positive effects in a concentration-dependent manner.

H_2_S is a gaseous signaling molecule that plays a dual role in human physiology [5]. Endogenously produced by host cells and gut bacteria, H_2_S has been implicated in various processes, including regulating inflammation, maintaining vascular tone, and the modulation of the immune system [6]. Its concentration-dependent effects render H_2_S a double-edged sword: while low concentrations of H_2_S are associated with cytoprotective and anti-inflammatory effects, high levels of H_2_S have been linked to cellular toxicity, tissue damage, and inflammatory processes [7]. This dichotomy is particularly relevant in the context of neurodegenerative diseases such as Alzheimer’s disease (AD) and Parkinson’s disease (PD), where disruptions in gut microbiota, known as dysbiosis, can lead to abnormal H_2_S production and the subsequent worsening of neuroinflammatory processes (Figure 1) [8].

Our manuscript provides a novel perspective by exploring microbiota-derived H_2_S as a critical modulator of the gut–brain axis, with implications for AD and PD. Unlike previous studies that focused on the individual roles of H_2_S, our manuscript integrates its dual effects on gut and blood–brain barrier (BBB) integrity, glial cell dysfunction, and therapeutic strategies involving probiotics, prebiotics, and dietary interventions. This holistic approach addresses key knowledge gaps and lays the foundation for novel therapeutic paradigms targeting H_2_S dysbiosis.

Dysbiosis refers to the imbalance in the composition and function of the gut microbiota, which can arise due to various factors such as diet, aging, infection, and antibiotics [9]. In neurodegenerative diseases, dysbiosis has been shown to affect the gut–brain axis, leading to impaired communication between the gut and the central nervous system (CNS) [10]. Alterations in gut microbial composition can result in changes in the production of microbial metabolites, including H_2_S, that influence glial cell function, neuroinflammation, and neuronal survival. In particular, the excessive production of H_2_S by sulfate-reducing bacteria (SRB) in the gut has been implicated in gut barrier dysfunction, systemic inflammation, and the increased permeability of the BBB, contributing to AD and PD [11].

SRBs are a significant subset of the gut microbiota known for their ability to utilize sulfate as a terminal electron acceptor, producing H_2_S as a metabolic byproduct. Commonly found SRBs in the human gut include *Desulfovibrio desulfuricans*, *Desulfovibrio vulgaris*, and *Desulfovibrio indonesiensis*. Elevated SRB populations are observed in patients with inflammatory bowel diseases (IBDs), such as ulcerative colitis (UC), where they have been correlated with active disease states. The presence of SRB in the gut becomes clinically relevant due to the production of H_2_S, with cytotoxic properties, by inhibiting butyrate oxidation in colonocytes, disrupting intestinal epithelial cell metabolism. This disruption of cellular functions, compounded with H_2_S’s ability to impair the protective mucus barrier of the intestines, allows for closer bacterial contact with the epithelial layer, thereby exacerbating inflammatory processes. Studies indicate that H_2_S influences immune responses and tissue architecture within the colon. For instance, SRB colonization in experimental models has led to the increased infiltration of immune cells in the gut lining and activation of T helper (Th17) and regulatory T (Treg) cells, both critical in managing mucosal immunity and inflammation. Additionally, SRB-related H_2_S may potentiate neutrophil recruitment and migration, driving further tissue damage and inflammation. SRB populations like *Desulfovibrio* spp. contribute to this pathogenic cycle by enhancing inflammation and perpetuating immune dysregulation within the gut, highlighting the importance of microbial composition and H_2_S modulation in managing gut health and diseases [12].

**Figure 2:**
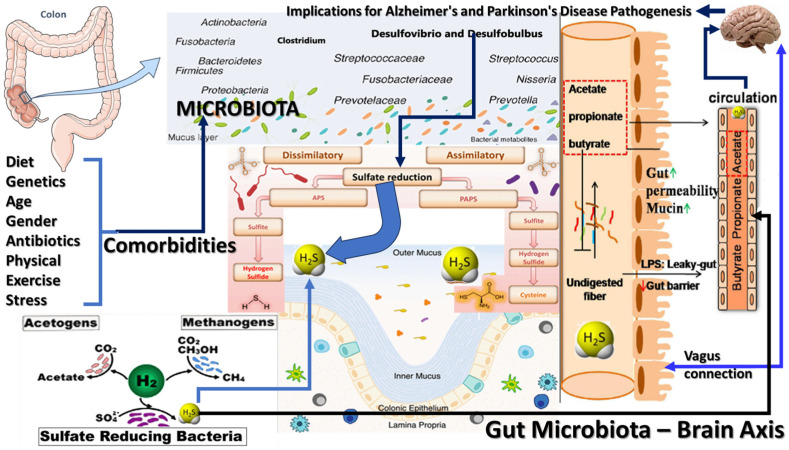
Illustrative interplay between diet, gut microbiota, and their metabolites in the context of neurodegenerative diseases, particularly Alzheimer’s and Parkinson’s. Diet shapes the gut microbial composition, including diverse groups such as Clostridium, Desulfovibrio, and Prevotella, responsible for metabolic activities with systemic impacts. Microbes metabolize dietary components into various products, including hydrogen sulfide (H_2_S) via sulfate reduction and short-chain fatty acids (SCFAs) like acetate, propionate, and butyrate from fiber fermentation. These metabolites influence gut permeability and barrier integrity, with butyrate supporting the gut lining. At the same time, excess H_2_S may compromise it, leading to a “leaky gut” and the release of inflammatory lipopolysaccharides (LPSs) into circulation. SCFAs and other microbial metabolites reach the brain through the bloodstream and vagus nerve, potentially promoting neuroinflammation—a known factor in neurodegeneration. The figure highlights how disruptions in gut microbiota balance can affect brain health, linking gut dysbiosis and altered barrier function to neurodegenerative disease risk and suggesting that therapeutic interventions targeting the gut may help mitigate these diseases.

The gut–brain axis encompasses multiple communication pathways (Figure 2), including the vagus nerve, the immune system, and the circulatory system [13]. Microbial metabolites such as H_2_S, SCFAs, and neurotransmitter precursors can traverse the gut barrier and directly influence CNS function [14]. The vagus nerve, which provides a direct neural link between the gut and the brain, plays a crucial role in communicating signals from the gut to brain regions involved in mood regulation, cognition, and motor control [15]. Dysregulation of the gut–brain axis, mainly due to alterations in microbial metabolite production, can disrupt this communication, leading to neuroinflammatory responses and glial cell activation, hallmarks of neurodegenerative diseases [16].

Neuroinflammation, a critical process in developing neurodegenerative diseases, involves activating glial cells, including astrocytes and microglia, which are responsible for maintaining CNS homeostasis and responding to injury [17]. In AD and PD, glial cells become activated in response to the accumulation of misfolded proteins, such as amyloid-beta in AD and alpha-synuclein in PD [18]. This activation releases pro-inflammatory cytokines, reactive oxygen species (ROS), and nitric oxide (NO), contributing to neuronal damage and synaptic dysfunction [19]. Microbial metabolites, including H_2_S, have been shown to modulate glial cell activity, with excessive H_2_S production exacerbating glial activation and promoting neuroinflammation [20].

Dysbiosis and altered H_2_S production in AD are associated with increased neuroinflammation, amyloid plaque formation, and synaptic dysfunction [21]. Studies have demonstrated that changes in gut microbiota composition, particularly the overgrowth of SRB, lead to elevated H_2_S levels in the gut, which can compromise the integrity of the gut barrier [22,23]. This action allows pro-inflammatory molecules and microbial products to enter the systemic circulation and reach the brain, triggering microglial activation and promoting amyloid-beta deposition. The resulting neuroinflammatory environment accelerates the progression of AD and contributes to cognitive decline [24].

Similarly, in PD, gut dysbiosis has been linked to gastrointestinal dysfunction, one of the earliest non-motor symptoms of the disease [25]. The gut microbiota of PD patients is often characterized by decreased beneficial bacteria and increased SRB, leading to abnormal H_2_S production [26]. Excessive H_2_S in the gut has been shown to impair gut barrier function, leading to the translocation of bacterial endotoxins and inflammatory mediators into the bloodstream [27]. These factors can cross the BBB and induce neuroinflammation, contributing to the accumulation of alpha-synuclein aggregates and the degeneration of dopaminergic neurons in the substantia nigra, a hallmark of PD [28].

The role of glial cells in neurodegenerative diseases has garnered considerable attention, particularly in their interaction with gut-derived metabolites [29]. Microglia, the resident immune cells of the CNS, are highly sensitive to changes in the gut microbiota and its metabolic products. Under normal conditions, microglia maintain CNS homeostasis by clearing debris, regulating synaptic plasticity, and modulating the immune response. However, in dysbiosis and excessive microbial H_2_S production, the microglial function becomes dysregulated, leading to chronic activation and the release of neurotoxic molecules. This sustained activation contributes to neurodegeneration by promoting neuronal death and synaptic dysfunction [30].

Astrocytes, another type of glial cell, also play a crucial role in maintaining CNS homeostasis and supporting neuronal function. Astrocytes regulate the extracellular environment, modulate synaptic transmission, and provide metabolic support to neurons. Astrocytes become reactive in response to neuroinflammation in neurodegenerative diseases and produce pro-inflammatory cytokines and ROS, further contributing to neuronal damage [31]. Microbial H_2_S has been shown to influence astrocyte function, with high levels of H_2_S exacerbating astrocyte reactivity and promoting neuroinflammation. The interaction between astrocytes and microglia in the context of dysbiosis and altered H_2_S production is a key factor in the progression of neurodegenerative diseases [32].

One central mechanism by which H_2_S contributes to neurodegeneration is through its impact on the BBB [33]. The BBB is a critical barrier that protects the brain from harmful substances in the bloodstream while allowing the selective transport of essential nutrients and molecules. Dysbiosis-induced changes in H_2_S production can compromise BBB integrity, allowing inflammatory mediators, microbial products, and other neurotoxic molecules to enter the brain. Once in the CNS, these factors contribute to neuroinflammation, glial activation, and neuronal dysfunction, all characteristic features of AD and PD [34].

The therapeutic potential of targeting the gut microbiota to modulate H_2_S production and restore the gut–brain axis function has become an area of intense research. Probiotics, live microorganisms that confer health benefits to the host, and prebiotics, non-digestible compounds that promote the growth of beneficial bacteria, have been proposed as potential interventions to mitigate dysbiosis and reduce neuroinflammation in neurodegenerative diseases [35]. Studies have shown that probiotic and prebiotic supplementation can restore microbial balance, reduce H_2_S production, and improve gut barrier integrity, attenuating neuroinflammatory responses and promoting neuroprotection [36,37].

In addition to probiotics and prebiotics, other therapeutic strategies targeting the gut–brain axis have been explored. Fecal microbiota transplantation (FMT), which involves the transfer of gut microbiota from a healthy donor to a recipient with dysbiosis, has shown promise in restoring microbial diversity and reducing neuroinflammation in preclinical models of AD and PD. By reintroducing beneficial microbial species and reducing the abundance of SRB, FMT can modulate H_2_S production and improve the gut–brain axis function, offering a novel therapeutic approach for neurodegenerative diseases [38].

Dietary interventions that modulate the gut microbiota and reduce H_2_S production have also been investigated as potential therapeutic strategies for neurodegenerative diseases. Diets rich in fiber and polyphenols, which promote the growth of beneficial bacteria and reduce the abundance of SRB, have been shown to improve gut barrier function and reduce neuroinflammation in animal models of AD and PD. These findings suggest that dietary modifications could effectively manage dysbiosis and its associated neuroinflammatory consequences in neurodegenerative diseases [39,40,41].

This study aims to investigate the dual role of microbiota-derived H_2_S as both a protective and harmful metabolite in the gastrointestinal tract and its broader systemic implications and to explore how H_2_S influences gut homeostasis, neuroinflammation, and neurodegeneration, particularly in Alzheimer’s and Parkinson’s diseases. It will also assess the impact of dysbiosis-driven H_2_S production on gut and blood–brain barrier integrity and examine H_2_S interactions with glial cells in modulating neuroinflammation and neuronal survival. Additionally, this study seeks to identify therapeutic interventions, such as probiotics and prebiotics, to modulate H_2_S production, restore homeostasis, and mitigate disease progression while expanding the understanding of the gut–brain axis in neurodegenerative pathogenesis.

## 2. Methodology

This narrative review aims to collect and synthesize evidence on how microbiota-derived H_2_S modulates the gut–brain axis and its implications for the pathogenesis of AD and PD. The review included studies focusing on human or animal models related to AD, PD, or general neurodegenerative mechanisms involving the gut–brain axis. Eligible studies explored microbiota-derived H_2_S production and its roles in gut homeostasis, neuroinflammation, gut–brain axis function, and neurodegenerative diseases. Outcomes of interest include the modulation of the gut–brain axis by H_2_S, its effects on neurodegeneration, neuroinflammation, the integrity of the gut and blood–brain barriers, and glial cell activity in AD and PD. Only reviews, original research, randomized controlled trials, observational studies, cohort studies, in vivo and in vitro experiments, and mechanistic studies were considered. At the same time, editorials, case reports, and conference abstracts were excluded.

A comprehensive search was conducted to identify relevant studies across multiple databases, including PubMed, Web of Science, Scopus, Nature, and Google Scholar. The search strategy included a combination of keywords and Medical Subject Headings (MeSH) terms such as “Microbiota”, “Hydrogen Sulfide” OR “H_2_S”, “Gut-Brain Axis”, “Alzheimer’s Disease”, “Parkinson’s Disease”, “Neuroinflammation”, “Dysbiosis”, “Glial cells”, and “Probiotics” OR “Prebiotics”. Boolean operators (AND, OR) were applied to refine the search, and truncation (*) was used to capture all relevant term variations.

A qualitative synthesis was conducted, providing a detailed narrative description of the findings, focusing on key themes such as the role of microbiota-derived H_2_S in modulating the gut–brain axis, the impact of dysbiosis on H_2_S production and its effects on gut and blood–brain barrier integrity, and the influence of H_2_S on neuroinflammation, glial cell activation, and neurodegeneration. As this study involves a narrative review of published data, no ethical approval was required.

## 3. Results

### 3.1. H_2_S as a Protective Agent in Gut Homeostasis

**Figure 3:**
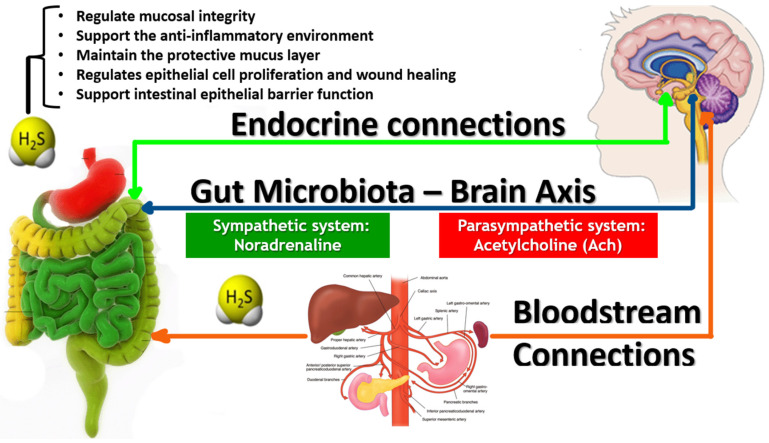
Bidirectional gut–brain axis, highlighting interactions among the hypothalamic–pituitary–adrenal (HPA) axis, gut microbiota, circulatory system, and neural pathways. The HPA axis produces cortisol, affecting immune cells, gut epithelium, and enteric muscles. Gut microbes modulate brain function via metabolites like short-chain fatty acids (SCFAs), neurotransmitters, and tryptophan metabolism. H_2_S was found to regulate mucosal integrity, support the anti-inflammatory environment of the gut, and maintain the protective mucus layer.

Numerous studies highlighted the protective role of H_2_S at physiological levels, contributing to gut homeostasis and overall gastrointestinal health (Figure 3). At low concentrations, H_2_S was found to regulate mucosal integrity, support the anti-inflammatory environment of the gut, and maintain the protective mucus layer [42]. It also regulates epithelial cell proliferation and wound healing, promotes intestinal epithelial barrier function, and inhibits pathogenic bacteria’s adhesion to the gut lining [43].

Moreover, H_2_S protects gut barrier integrity by preserving tight junction proteins (e.g., claudin, occludin, and zonula occludens-1) and preventing microbial toxins’ translocation into the systemic circulation. These findings underscore the importance of H_2_S in regulating immune responses, preventing microbial invasion, and maintaining gut health overall [44].

### 3.2. Dichotomous Nature of H_2_S in Inflammation and Gut Permeability

Despite its protective role at physiological concentrations, studies demonstrated that elevated H_2_S levels, often associated with dysbiosis, harm gut health. Excessive production of H_2_S, particularly by SRB such as *Desulfovibrio* species, was linked to increased gut permeability, often referred to as “leaky gut” [45]. Elevated H_2_S levels disrupt the epithelial barrier, translocating pro-inflammatory molecules (e.g., lipopolysaccharides) into the bloodstream, triggering systemic inflammation [46].

In animal models of dysbiosis, high concentrations of microbiota-derived H_2_S were associated with the degradation of gut mucins, weakening the mucus layer and further compromising gut integrity. These findings suggest that H_2_S plays a dual role: it supports gut health at low concentrations but contributes to inflammatory responses and gut barrier dysfunction when produced in excess [47].

### 3.3. Impact of H_2_S on Neuroinflammation and the Gut–Brain Axis

Neuroinflammation is a key mechanism through which H_2_S influences the gut–brain axis. Chronic neuroinflammation is a hallmark of disease progression in both AD and PD, often driven by the activation of glial cells and the accumulation of misfolded proteins (e.g., amyloid-beta in AD and alpha-synuclein in PD). In studies examining neurodegenerative models, elevated H_2_S levels were associated with the increased expression of neuroinflammatory markers, including IL-1β, TNF-α, and IL-6, known to exacerbate neuronal damage. The inhibition of H_2_S production or the modulation of gut microbiota to lower H_2_S levels has been shown to reduce these markers, highlighting the potential therapeutic value of targeting H_2_S in neuroinflammatory pathways [48].

The review identified several key studies linking dysregulated H_2_S production to alterations in the gut–brain axis, mainly through promoting neuroinflammation. Excessive H_2_S production affected both peripheral and central immune responses, leading to the chronic activation of glial cells, such as microglia and astrocytes, in the central nervous system (CNS). This chronic activation contributes to the neurodegenerative processes. In both human and animal studies, elevated H_2_S levels were correlated with increased markers of neuroinflammation, including elevated levels of pro-inflammatory cytokines (e.g., IL-1β, IL-6, TNF-α) in cerebrospinal fluid and brain tissue. These cytokines are known to exacerbate neuronal damage and are central to the neuroinflammatory cascades observed in neurodegenerative diseases [49,50,51,52,53].

### 3.4. H_2_S and Blood–Brain Barrier Dysfunction

The blood–brain barrier plays a crucial role in maintaining CNS homeostasis by selectively regulating the passage of substances from the bloodstream into the brain. Similar to its effects on the gut barrier, H_2_S can influence the permeability of the BBB. Under normal conditions, H_2_S may help preserve the BBB’s integrity by regulating oxidative stress and inflammatory processes. However, elevated levels of microbiota-derived H_2_S, particularly in the presence of systemic inflammation, can compromise the BBB [54].

When the BBB is disrupted, harmful substances such as pro-inflammatory cytokines, microbial toxins, and activated immune cells can infiltrate the CNS. This breach in the BBB is a critical factor in neurodegenerative diseases like AD and PD, where neuroinflammation accelerates the degeneration of neurons. In animal models, high levels of microbiota-derived H_2_S were associated with increased expressions of endothelial adhesion molecules, which facilitate the infiltration of immune cells into the brain. This infiltration is a critical factor in neuroinflammation and further supports the detrimental role of excessive H_2_S production in neurodegeneration [55,56,57].

### 3.5. H_2_S and Glial Cell Dysfunction in Neurodegeneration

At physiological levels, H_2_S exerts anti-inflammatory effects on glial cells, limiting excessive microglial activation and promoting the resolution of inflammation. This neuroprotective role of H_2_S helps prevent chronic neuroinflammatory conditions that could damage neurons [58,59]. This review highlights the role of microbiota-derived H_2_S in modulating glial cell activity, with studies demonstrating that dysregulated H_2_S production exacerbates glial cell dysfunction [60]. In AD and PD, glial cells (microglia and astrocytes) play a central role in maintaining CNS homeostasis and responding to neuronal injury. However, the excessive activation of these cells, driven in part by elevated H_2_S levels, contributes to a sustained inflammatory environment in the brain, leading to progressive neuronal loss [61].

In vitro studies showed that H_2_S can directly modulate glial cell activation. At physiological concentrations, H_2_S exerts neuroprotective effects by limiting microglial activation and promoting the resolution of inflammation. However, at higher concentrations, it promotes a pro-inflammatory phenotype in glial cells. It is characterized by releasing neurotoxic molecules such as ROS and nitric oxide (NO), further accelerating neurodegeneration [62].

### 3.6. Interaction with Neural Signaling via the Vagus Nerve

The vagus nerve is a significant component of the parasympathetic nervous system and provides a direct communication pathway between the gut and the brain. H_2_S can modulate vagal signaling by affecting the release of neuroactive substances from the gut. For example, H_2_S may influence the production of neurotransmitters such as serotonin, dopamine, and gamma-aminobutyric acid (GABA), all of which play crucial roles in CNS function and mood regulation [63,64,65].

In the context of neurodegenerative diseases, alterations in H_2_S levels may lead to the dysregulation of vagal signaling, contributing to symptoms such as mood disturbances, cognitive decline, and motor dysfunction. The gut–brain signaling mediated by the vagus nerve may be particularly important in the early stages of PD, where gastrointestinal symptoms often precede the onset of motor symptoms by several years. Dysregulated H_2_S production in the gut may be an early contributor to this process, suggesting that the therapeutic modulation of H_2_S levels could have far-reaching effects on gut and brain health [66,67].

### 3.7. Modulation of Mitochondrial Function and Oxidative Stress

H_2_S is also involved in modulating mitochondrial function, essential for maintaining cellular energy balance and managing oxidative stress. Within the gut and CNS, H_2_S regulates mitochondrial biogenesis and functions by influencing electron transport and ATP production. At low levels, H_2_S enhances mitochondrial function and reduces oxidative stress, thereby protecting neurons from damage [68].

However, excessive H_2_S can inhibit mitochondrial respiratory enzymes and increase the production of ROS, leading to oxidative stress, mitochondrial dysfunction, and neuronal injury. This effect is particularly relevant in neurodegenerative diseases, where mitochondrial dysfunction is a known contributor to disease progression. H_2_S-induced oxidative stress further exacerbates the inflammatory environment in the brain, amplifying neurodegeneration [69].

### 3.8. Microbial Metabolite Interactions and Neurotransmitter Regulation

Microbiota-derived H_2_S interacts with other microbial metabolites, such as short-chain fatty acids (SCFAs) and tryptophan metabolites, which play critical roles in gut–brain communication [70]. These metabolites can regulate neurotransmitter production and modulate brain function. Dysbiosis-associated increases in H_2_S may alter the balance of these metabolites, leading to disruptions in neurotransmitter synthesis and signaling [71,72,73].

For example, SCFAs have anti-inflammatory effects and can protect the integrity of the gut and blood–brain barriers. High levels of H_2_S may interfere with the beneficial effects of SCFAs by promoting inflammatory responses and barrier dysfunction [74]. Additionally, tryptophan metabolism by gut bacteria is a key pathway for producing serotonin, a neurotransmitter implicated in mood regulation and cognitive function. Dysregulated H_2_S production may affect tryptophan metabolism, contributing to neurotransmitter imbalances commonly seen in AD and PD [75].

## 4. Age, Gender, and Comorbidities in Gut Microbiota Composition and H_2_S Production

### 4.1. Age-Related Changes in Gut Microbiota Composition and H_2_S Production

The aging process is a critical factor influencing the composition and function of the gut microbiota and the production of microbiota-derived metabolites such as H_2_S. As individuals age, profound changes occur within the gut–brain axis, impacting immune responses, barrier integrity, and neurological health. These alterations have significant implications for the development and progression of neurodegenerative diseases such as AD and PD. Understanding how age-related changes in microbiota-derived H_2_S influence the gut–brain axis provides crucial insights into the mechanisms underlying neurodegeneration [76].

Aging is associated with a decline in the diversity and stability of the gut microbiota, often called “microbiota aging”. In elderly individuals, beneficial bacterial populations such as *Lactobacillus* and *Bifidobacterium* species tend to decline. In contrast, opportunistic and pathogenic bacteria, including SRB such as *Desulfovibrio*, may increase in abundance. This shift in microbial composition can lead to the increased production of microbial metabolites like H_2_S, which has protective and harmful effects depending on its concentration [77]. The rise in SRB populations during aging is particularly concerning, as these bacteria produce higher levels of H_2_S by reducing sulfur compounds. Increased H_2_S production in the aging gut can exacerbate inflammation and impair gut barrier integrity, contributing to the translocation of pro-inflammatory molecules into the bloodstream. This systemic inflammation, in turn, influences the gut–brain axis, playing a role in neurodegenerative processes [78].

With age, the integrity of the gut barrier often deteriorates due to several factors, including reduced tight junction protein expression, immune dysregulation, and increased oxidative stress. These factors contribute to the “leaky gut”, where the barrier becomes more permeable to harmful substances, including microbial endotoxins and inflammatory cytokines [79]. Excessive H_2_S production by age-associated dysbiotic microbiota further compromises gut barrier function. While at physiological levels, H_2_S helps maintain barrier integrity by promoting mucin secretion and tight junction stabilization, elevated levels lead to the degradation of the mucus layer and the disruption of tight junctions, allowing inflammatory molecules to enter the systemic circulation. The increased permeability and systemic inflammation associated with this gut barrier dysfunction have been linked to the development of neuroinflammation and neurodegenerative diseases [80,81].

### 4.2. Gender Differences in Gut Microbiota Composition and H_2_S Production

Gut microbiota composition differs between men and women, influenced by factors such as sex hormones (e.g., estrogen, progesterone, testosterone), immune responses, and lifestyle behaviors. Gender plays a significant role in modulating the production and effects of microbiota-derived H_2_S, influencing the gut–brain axis and contributing to the pathogenesis of neurodegenerative diseases like AD and PD. Differences in gut microbiota composition, hormone regulation, immune responses, and barrier integrity between men and women lead to distinct patterns of H_2_S dysregulation [82,83].

Estrogen has protective effects on gut barrier integrity, enhancing the expression of tight junction proteins and reducing gut permeability. These protective effects may help mitigate the harmful effects of excessive H_2_S production in women by maintaining gut barrier function and preventing the translocation of harmful substances like lipopolysaccharides (LPSs) and pro-inflammatory cytokines into the bloodstream. As a result, women may be somewhat protected from the gut barrier dysfunction associated with elevated H_2_S levels, at least until hormonal changes occur during menopause [84,85].

Estrogen has been shown to enhance BBB integrity, reduce permeability, and protect the brain from inflammatory mediators. This protective effect may limit the impact of excessive H_2_S on the BBB in women, reducing the translocation of inflammatory molecules from the gut into the brain. Consequently, women may experience less H_2_S-driven neuroinflammation and BBB disruption, especially during their reproductive years [86]. However, after menopause, the decline in estrogen levels may reduce this protective effect, leading to increased BBB permeability and heightened susceptibility to H_2_S-induced neuroinflammation. This phenomenon may partly explain why postmenopausal women experience a higher risk of developing AD, as the combination of gut dysbiosis, increased H_2_S production, and BBB disruption may accelerate neurodegenerative processes [87,88].

In women, higher levels of *Bifidobacterium* and *Lactobacillus* species are commonly observed, associated with the lower production of harmful metabolites, including H_2_S. However, fluctuations in hormonal levels, such as during the menstrual cycle, pregnancy, and menopause, can influence gut microbial composition and metabolism. For example, estrogen has been shown to promote the growth of beneficial bacterial species less likely to produce excessive H_2_S. During menopause, however, the decline in estrogen levels may lead to shifts in the gut microbiota, including an increased abundance of SRB like *Desulfovibrio*, which produces higher levels of H_2_S [89].

In men, higher testosterone levels have been associated with a less protective gut barrier, potentially increasing susceptibility to H_2_S-induced gut barrier dysfunction. The higher H_2_S production from SRBs and reduced gut barrier integrity may lead to greater gut permeability (“leaky gut”) in men, contributing to systemic inflammation and promoting neuroinflammatory processes linked to neurodegenerative diseases like PD [90,91].

Men tend to have a higher abundance of SRBs than women, potentially resulting in more outstanding productions of H_2_S. Higher testosterone levels may partly influence this gender-specific microbial composition in men, which has been linked to alterations in gut microbiota that favor H_2_S production. These differences in microbial H_2_S production may contribute to the heightened risk of certain gastrointestinal and neuroinflammatory conditions observed in men. Moreover, men may experience more pronounced H_2_S-related disruptions in the gut–brain axis, influencing the progression of neurodegenerative diseases such as PD, which has a higher prevalence in males [92,93].

Men, with their typically lower estrogen levels, may have less protection against BBB disruption, especially in the presence of elevated H_2_S. The combination of higher gut-derived H_2_S production reduced gut and BBB integrity, and more pronounced neuroinflammatory responses may contribute to men’s greater vulnerability to PD, where neuroinflammation plays a central role in dopaminergic neuron loss [94].

Given the gender differences in microbiota composition, immune responses, and hormone regulation, gender-specific therapeutic approaches may be necessary for effectively modulating H_2_S and mitigating its impact on neurodegenerative diseases [95]. Hormone replacement therapy (HRT) could also help maintain gut and BBB integrity by compensating for the loss of estrogen. Probiotics, prebiotics, and dietary interventions designed to reduce SRB abundance and promote beneficial bacteria may mainly effectively reduce H_2_S production and neuroinflammation in women [96].

In men, interventions targeting the higher baseline levels of SRBs and H_2_S production may be critical. Anti-inflammatory therapies and probiotics or dietary approaches that specifically inhibit SRBs could help reduce the harmful effects of excessive H_2_S on the gut–brain axis. Additionally, therapies aimed at enhancing gut barrier integrity and protecting the BBB may be particularly beneficial for men, who are more susceptible to H_2_S-driven barrier dysfunction and neuroinflammation [97,98,99].

### 4.3. Other Comorbidities, Gut Microbiota Composition and H_2_S Production

Comorbidities, particularly those related to metabolic, cardiovascular, gastrointestinal, and immune systems, significantly influence the production and effects of microbiota-derived H_2_S on the gut–brain axis. These comorbidities often exacerbate the pathogenesis of neurodegenerative diseases like AD and PD by disrupting gut homeostasis, increasing systemic inflammation, and promoting neuroinflammation [100].

Metabolic disorders, including obesity, type 2 diabetes (T2D), and metabolic syndrome, are strongly associated with alterations in gut microbiota composition and function, leading to dysbiosis and changes in H_2_S production. These conditions exacerbate systemic inflammation and increase the risk of neurodegenerative diseases by disrupting the gut–brain axis. Type 2 diabetes (T2D) is characterized by chronic hyperglycemia, insulin resistance, and systemic inflammation, all linked to changes in gut microbiota composition. T2D is associated with increased H_2_S production due to dysbiosis, particularly overrepresenting SRBs in the gut. Elevated H_2_S exacerbates insulin resistance by disrupting gut barrier integrity and promoting systemic inflammation. The gut–brain axis plays a critical role in linking T2D and neurodegeneration. Insulin resistance in the CNS is associated with cognitive decline and increased amyloid-beta deposition, key features of AD. Furthermore, increased H_2_S production in T2D contributes to neuroinflammation by promoting the activation of microglia and astrocytes, which release pro-inflammatory mediators that damage neurons. This inflammation is particularly harmful in the hippocampus, a brain region essential for memory and cognition severely affected by AD [101].

Obesity is linked to a pro-inflammatory state and changes in the gut microbiota that promote the overgrowth of sulfate-reducing bacteria (SRB), such as *Desulfovibrio* species, which produce H_2_S. Excessive H_2_S production in obese individuals contributes to gut barrier dysfunction, allowing the translocation of lipopolysaccharides (LPSs) and other bacterial endotoxins into the bloodstream. This systemic inflammation affects metabolic tissues and the CNS, promoting neuroinflammation and contributing to the development of AD and PD. In obesity, the elevated levels of pro-inflammatory cytokines, such as IL-6 and TNF-α, further exacerbate the disruption of the gut–brain axis. This chronic low-grade inflammation increases oxidative stress and accelerates neuronal damage, creating a conducive environment for neurodegeneration. Moreover, adipose tissue dysfunction in obesity releases free fatty acids and inflammatory molecules that further amplify neuroinflammatory responses, particularly in brain regions affected by AD and PD [102,103,104,105].

Cardiovascular diseases, including hypertension, atherosclerosis, and heart failure, significantly impact gut microbiota composition and H_2_S production, contributing to neuroinflammation and neurodegeneration through multiple pathways. Hypertension is closely associated with gut dysbiosis, where an imbalance in microbial populations leads to increased H_2_S production by SRBs. This dysregulation contributes to endothelial dysfunction and inflammation, both exacerbating hypertension and increasing the BBB permeability. A compromised BBB allows the translocation of inflammatory molecules and microbial metabolites, such as H_2_S, into the CNS, leading to neuroinflammation. In hypertensive individuals, the interaction between dysregulated H_2_S and vascular endothelial cells contributes to oxidative stress and ROS formation. This oxidative damage not only affects the cardiovascular system but also promotes neuroinflammation, increasing the risk of neurodegenerative diseases like AD and PD. Elevated H_2_S levels in the context of hypertension may also impair cerebral blood flow, further contributing to neuronal dysfunction and cognitive decline [101,106].

Atherosclerosis, characterized by chronic vascular inflammation and plaque formation in arteries, is linked to gut microbiota dysbiosis and altered microbial metabolite production, including H_2_S. Increased H_2_S levels contribute to systemic inflammation and endothelial dysfunction, promoting plaque instability and increasing the risk of cardiovascular events. The systemic inflammation associated with atherosclerosis extends to the CNS, where H_2_S modulates the activation of glial cells, particularly microglia, leading to neuroinflammation. This chronic inflammatory state accelerates neuronal damage and promotes the aggregation of misfolded proteins, such as amyloid-beta in AD and alpha-synuclein in PD. The vascular dysfunction in atherosclerosis also affects cerebral blood flow, reducing oxygen and nutrient delivery to the brain and exacerbating neurodegenerative processes [107,108,109].

Gastrointestinal (GI) disorders such as inflammatory bowel disease (IBD), irritable bowel syndrome (IBS), and small intestinal bacterial overgrowth (SIBO) are associated with significant alterations in gut microbiota and H_2_S production [110]. These conditions disrupt the gut–brain axis and increase the risk of neuroinflammation and neurodegeneration. IBD, including Crohn’s disease and ulcerative colitis, is characterized by chronic inflammation of the gastrointestinal tract. Dysbiosis in IBD is associated with increased populations of SRBs, leading to elevated H_2_S production. Excessive H_2_S exacerbates intestinal inflammation and contributes to gut barrier dysfunction, allowing microbial products to enter the bloodstream and reach the brain. The systemic inflammation and increased permeability associated with IBD extend to the CNS, where the inflammatory mediators and microbial metabolites such as H_2_S contribute to neuroinflammation. In patients with IBD, neuroinflammatory processes are linked to an increased risk of cognitive decline and neurodegenerative diseases like AD and PD. Chronic gut inflammation in IBD may also prime microglia in the CNS, making them more responsive to inflammatory signals and increasing the likelihood of neurodegeneration. IBS is a functional gastrointestinal disorder characterized by altered gut motility, visceral hypersensitivity, and dysbiosis. In IBS, the gut microbiota often shows an increased abundance of SRBs, resulting in elevated H_2_S levels. This increase in H_2_S can disrupt gut barrier integrity, leading to a heightened immune response and systemic inflammation [111,112].

Patients with IBS frequently report anxiety, depression, and cognitive symptoms, which are linked to gut–brain axis dysregulation. Elevated H_2_S levels may play a role in this dysregulation by modulating neural and immune signaling pathways. Although the exact mechanisms are still under investigation, the relationship between elevated H_2_S, gut inflammation, and CNS dysfunction suggests that IBS may increase the risk of neuroinflammatory conditions that contribute to AD and PD [113].

Immune-mediated disorders, including autoimmune diseases such as rheumatoid arthritis (RA) and systemic lupus erythematosus (SLE), are characterized by chronic inflammation and altered immune responses. These conditions affect the gut microbiota and H_2_S production, contributing to systemic and neuroinflammation [114,115].

RA is associated with gut dysbiosis, leading to increased H_2_S production and altered immune responses. Chronic inflammation in RA affects the joints and extends to the gut and CNS. Elevated H_2_S levels exacerbate gut barrier dysfunction, promoting the translocation of inflammatory mediators to the brain and increasing the risk of neuroinflammation. In RA, microglial activation in response to systemic inflammation contributes to neurodegenerative processes, particularly AD. The chronic immune activation and increased H_2_S production in RA may accelerate neuronal damage by promoting oxidative stress and mitochondrial dysfunction in neurons [116,117,118].

SLE, an autoimmune disorder characterized by widespread inflammation and tissue damage, is linked to gut dysbiosis and increased H_2_S production. Similar to RA, SLE involves chronic inflammation that disrupts the gut–brain axis. Elevated H_2_S levels contribute to gut barrier dysfunction and promote systemic inflammation, which can extend to the CNS. In SLE, the neuroinflammatory processes linked to increased H_2_S production may accelerate neurodegeneration by promoting glial cell activation and the release of pro-inflammatory cytokines. Patients with SLE are at increased risk of developing cognitive impairments and neurodegenerative diseases, likely due to the combined effects of chronic inflammation, dysbiosis, and H_2_S dysregulation [119,120,121].

Neuropsychiatric conditions, such as depression and anxiety, are associated with altered gut microbiota and the dysregulated production of microbial metabolites, including H_2_S. These conditions influence mood and cognition, affect the gut–brain axis, and may increase the risk of neurodegenerative diseases. Patients with depression and anxiety often exhibit dysbiosis, including increased H_2_S production. This dysregulation may exacerbate gut barrier dysfunction and promote systemic inflammation, contributing to neuroinflammatory processes in the brain. The chronic low-grade inflammation associated with depression and anxiety has been linked to the development of AD and PD, where neuroinflammation plays a central role in disease progression [122,123,124].

## 5. Therapeutic Targets and Interventions Related to H_2_S Modulation

Modulating microbiota-derived H_2_S offers promising therapeutic potential, particularly for neurodegenerative diseases, where dysregulated H_2_S levels contribute to gut–brain axis disruption, neuroinflammation, and neuronal damage. Effective interventions focus on regulating microbial H_2_S production and mitigating its harmful effects while harnessing its beneficial roles. The following sections outline various therapeutic strategies and targets aimed at H_2_S modulation.

### 5.1. Probiotics and Prebiotics

Probiotics and prebiotics are well-established interventions for modulating gut microbiota and have demonstrated potential in regulating H_2_S production, particularly by reducing the abundance of sulfate-reducing bacteria (SRB), such as *Desulfovibrio* species, responsible for excessive H_2_S production [125,126,127].

Probiotics are live microorganisms that confer health benefits when administered in adequate amounts. Certain probiotic strains, such as *Lactobacillus* and *Bifidobacterium* species, have been shown to restore gut microbial balance by inhibiting the growth of SRBs and promoting beneficial bacteria. By outcompeting SRBs for resources in the gut, probiotics can reduce H_2_S production and its associated toxic effects on the gut barrier [128,129].

Additionally, probiotics modulate gut permeability by enhancing the expression of tight junction proteins, thereby reducing gut barrier dysfunction (“leaky gut”) and limiting the translocation of harmful microbial products into the bloodstream. In neurodegenerative diseases such as AD and PD, probiotics have shown promise in reducing systemic inflammation, improving cognitive and motor functions, and protecting against neuroinflammation [130,131,132].

Prebiotics are non-digestible food components that promote the growth and activity of beneficial gut bacteria. Prebiotics such as inulin, fructooligosaccharides (FOSs), and resistant starch can increase the production of short-chain fatty acids (SCFAs), particularly butyrate, which helps maintain gut integrity and inhibit SRB proliferation [133,134].

Prebiotics promote a favorable gut environment and reduce microbial dysbiosis and the overproduction of H_2_S. The increase in SCFA levels also has anti-inflammatory effects in the gut and the CNS, as SCFAs are known to modulate immune responses and reduce neuroinflammation. These effects make prebiotics a valuable therapeutic option for managing neuroinflammation in AD and PD [135,136].

### 5.2. Dietary Modifications

Diet plays a significant role in shaping the gut microbiota composition and, consequently, the levels of microbial-derived metabolites, including H_2_S. Dietary interventions can modulate H_2_S production by influencing the substrates available for SRBs and promoting the growth of beneficial bacteria [137,138].

Sulfate-reducing bacteria derive H_2_S from sulfur-containing compounds such as sulfates, sulfites, and sulfur-containing amino acids (e.g., cysteine and methionine). A low-sulfur diet, which reduces the intake of these compounds, can limit the substrate availability for SRBs and consequently decrease H_2_S production. This dietary intervention may help reduce the harmful effects of excessive H_2_S on gut and BBB integrity, thereby alleviating systemic inflammation and neuroinflammation [139].

A diet rich in dietary fiber promotes the growth of beneficial gut bacteria that produce SCFAs while reducing the growth of SRBs. High-fiber foods, such as whole grains, legumes, fruits, and vegetables, support a healthy gut environment that reduces H_2_S production. Fiber fermentation by beneficial bacteria increases SCFA production, which helps to preserve gut barrier integrity, reduce inflammation, and enhance the anti-inflammatory effects on the CNS [140,141]. High-fiber diets have been linked to improved gut health and reduced risks of neuroinflammatory conditions. In neurodegenerative disease models, they have mitigated cognitive decline and motor dysfunction by promoting neuroprotective microbial metabolites and reducing harmful H_2_S levels [142].

### 5.3. Fecal Microbiota Transplantation (FMT)

Fecal microbiota transplantation (FMT) involves transferring gut microbiota from a healthy donor to a recipient with dysbiosis. FMT has shown promise in restoring microbial balance, reducing dysbiosis-associated H_2_S production and improving gut barrier function. In preclinical models of neurodegenerative diseases, FMT has demonstrated the potential to alleviate symptoms by reducing systemic and neuroinflammation. By restoring microbial diversity and reducing the abundance of SRBs, FMT can help modulate H_2_S levels, thereby protecting the gut–brain axis and mitigating the progression of AD and PD. However, further research and clinical trials are necessary to fully understand the long-term effects of FMT on H_2_S modulation and neurodegenerative diseases [143,144,145].

### 5.4. Pharmacological Inhibitors of H_2_S Production

Pharmacological interventions targeting H_2_S-producing enzymes or SRBs offer a more direct approach to modulating H_2_S levels. Enzymatic inhibitors or compounds that selectively target H_2_S-producing bacteria have been explored as potential therapeutic strategies for reducing the harmful effects of excessive H_2_S [146].

Host-derived H_2_S is produced by enzymes such as cystathionine γ-lyase (CSE) and cystathionine β-synthase (CBS). Inhibitors of these enzymes can reduce endogenous H_2_S production, which may be beneficial in conditions where excessive H_2_S contributes to neuroinflammation. Selective inhibitors of CSE and CBS are being explored for their potential to modulate H_2_S levels in inflammatory and neurodegenerative diseases [147]. However, care must be taken with enzyme inhibitors, as H_2_S plays protective roles at physiological levels, particularly in maintaining vascular homeostasis and modulating immune responses. Targeted inhibition should aim to reduce excessive H_2_S without compromising its beneficial effects [148]. Developing antibacterial agents that selectively inhibit SRBs could help reduce microbial H_2_S production. Such agents would ideally target SRB-specific metabolic pathways without disrupting the overall balance of the gut microbiota. Although these targeted approaches are still under investigation, the potential of the selective inhibition of SRBs represents a promising therapeutic strategy for reducing the harmful effects of microbiota-derived H_2_S in neurodegenerative diseases, offering optimism for future treatments [149].

### 5.5. Antioxidants and Anti-Inflammatory Agents

Given that excessive H_2_S promotes oxidative stress and inflammation, therapeutic strategies to counteract these effects have gained attention. Antioxidants and anti-inflammatory agents may mitigate the damaging effects of H_2_S on neurons and glial cells, particularly in neuroinflammation. Antioxidants such as N-acetylcysteine (NAC), glutathione, and vitamin C can scavenge ROS generated by excessive H_2_S. These compounds help protect neurons from oxidative damage and may reduce the pro-inflammatory effects of H_2_S in the CNS. In preclinical models, antioxidant therapy has been shown to alleviate neuroinflammation and improve cognitive function in neurodegenerative diseases [150,151].

Pharmacological agents inhibiting pro-inflammatory signaling pathways, such as NF-κB inhibitors, can reduce the inflammatory response of excessive H_2_S. By limiting the activation of glial cells and reducing the production of pro-inflammatory cytokines, these agents may help counteract the neuroinflammatory effects of dysregulated H_2_S levels. Non-steroidal anti-inflammatory drugs (NSAIDs) and other more selective anti-inflammatory agents are being explored for their potential in modulating neuroinflammation in AD and PD [152,153].

### 5.6. Combination Therapies

Given the multifactorial nature of H_2_S’s role in neurodegenerative diseases, combination therapies that target multiple aspects of H_2_S modulation may offer the most effective outcomes. For example, combining probiotics or prebiotics with dietary interventions, antioxidant therapy, or pharmacological inhibitors of H_2_S production may synergistically restore gut and brain homeostasis [154,155].

The modulation of microbiota-derived H_2_S represents a promising therapeutic strategy for addressing neuroinflammation and neurodegeneration, particularly in diseases such as Alzheimer’s and Parkinson’s. Probiotics, prebiotics, dietary modifications, fecal microbiota transplantation, and pharmacological inhibitors offer potential avenues for reducing excessive H_2_S production and mitigating its harmful effects. Additionally, antioxidants and anti-inflammatory agents provide complementary approaches to counteract the oxidative stress and inflammation associated with dysregulated H_2_S levels. Future research and clinical trials will be essential in optimizing these therapeutic interventions and developing effective treatments for neurodegenerative diseases by targeting H_2_S modulation [156].

## 6. Limitations and Gaps in Current Research

While the review identified a substantial body of evidence supporting the role of microbiota-derived H_2_S in modulating the gut–brain axis and contributing to neurodegeneration, several gaps remain. First, H_2_S levels or the exact mechanisms through which H_2_S modulates glial cell activity and promotes neuroinflammation require further elucidation. Second, human studies investigating the direct impact of H_2_S modulation on neurodegenerative outcomes are limited, necessitating more clinical trials in this area. Lastly, neurodegeneration needs further exploration of the interaction between H_2_S and other microbial metabolites, such as SCFAs and tryptophan metabolites [157,158].

## 7. Conclusions

Microbiota-derived H_2_S plays a critical dual role in modulating the gut–brain axis, with protective and harmful effects depending on its concentration. At physiological levels, H_2_S supports gut barrier integrity, regulates immune responses, and maintains homeostasis within the gut–brain axis. However, dysbiosis and comorbidities like metabolic disorders, cardiovascular diseases, and gastrointestinal conditions often lead to excessive H_2_S production, exacerbating gut permeability, BBB disruption, and systemic inflammation. This dysregulation promotes chronic neuroinflammation, primarily through activating glial cells, including microglia and astrocytes, which release pro-inflammatory cytokines and ROS, accelerating neuronal damage in neurodegenerative diseases such as AD and PD. Gender differences further influence H_2_S production, with estrogen offering protective effects in women by maintaining barrier integrity and limiting H_2_S-induced inflammation. At the same time, men with higher levels of sulfate-reducing bacteria may be more vulnerable to the harmful effects of H_2_S. Therapeutic interventions that modulate H_2_S production, including probiotics, prebiotics, dietary changes, and pharmacological inhibitors, offer promising strategies to restore microbial balance, improve gut–brain axis function, and mitigate neuroinflammation. Given the central role of H_2_S in disrupting gut and BBB integrity, targeting microbial H_2_S and restoring gut homeostasis are critical for preventing or slowing the progression of neurodegenerative diseases. These approaches must consider individual factors such as gender, age, and comorbidities to optimize therapeutic efficacy.
